# Case series: Congenital left ventricular diverticulum

**DOI:** 10.4103/0971-3026.69356

**Published:** 2010-08

**Authors:** Dharita Shah, C Prem Kumar, Mukesh S Shah, Mihir Baraiya

**Affiliations:** Department of Radio-Diagnosis, Seth Vadilal Sarabhai General Hospital, Ellisbridge, Ahmedabad - 380 016, Gujarat, India

**Keywords:** Left ventricular diverticulum, Cantrell’s pentalogy, CT angiography

## Abstract

Congenital left ventricular diverticulum is a rare cardiac malformation characterized by a localized outpouching from the cardiac chamber. The patient is usually asymptomatic. However, complications like embolism, infective endocarditis, arrhythmia and, rarely, rupture can be the initial presentation. Diagnosis can be established by USG, echocardiography, CT angiography, and MRI. We report here two neonates with congenital left ventricular apical diverticulum associated with epigastric hernia.

## Introduction

Congenital left ventricular diverticulum is a rare cardiac abnormality consisting of a localized outpouching from the free wall of the cardiac chamber. Commonly, this is from the left ventricular apex; however, non-apical diverticula also occur.

## Case Reports

Case 1

A 1-day-old newborn baby presented with epigastric pulsation and umbilical hernia and underwent routine postnatal investigation.

Antenatally, the 21-year-old mother, a known case of epilepsy on regular treatment, was diagnosed to have a baby with a ventricular septal defect (VSD). Fetal echo at the 7^th^month of intrauterine life showed normal atrioventricular concordance and normal relation of the great vessels with the ventricular chambers. A large subaortic VSD, a patent foramen ovale, and a large ductus arteriosus were also noted. Ventricular function was normal.

**Figure 1 (A,B):**
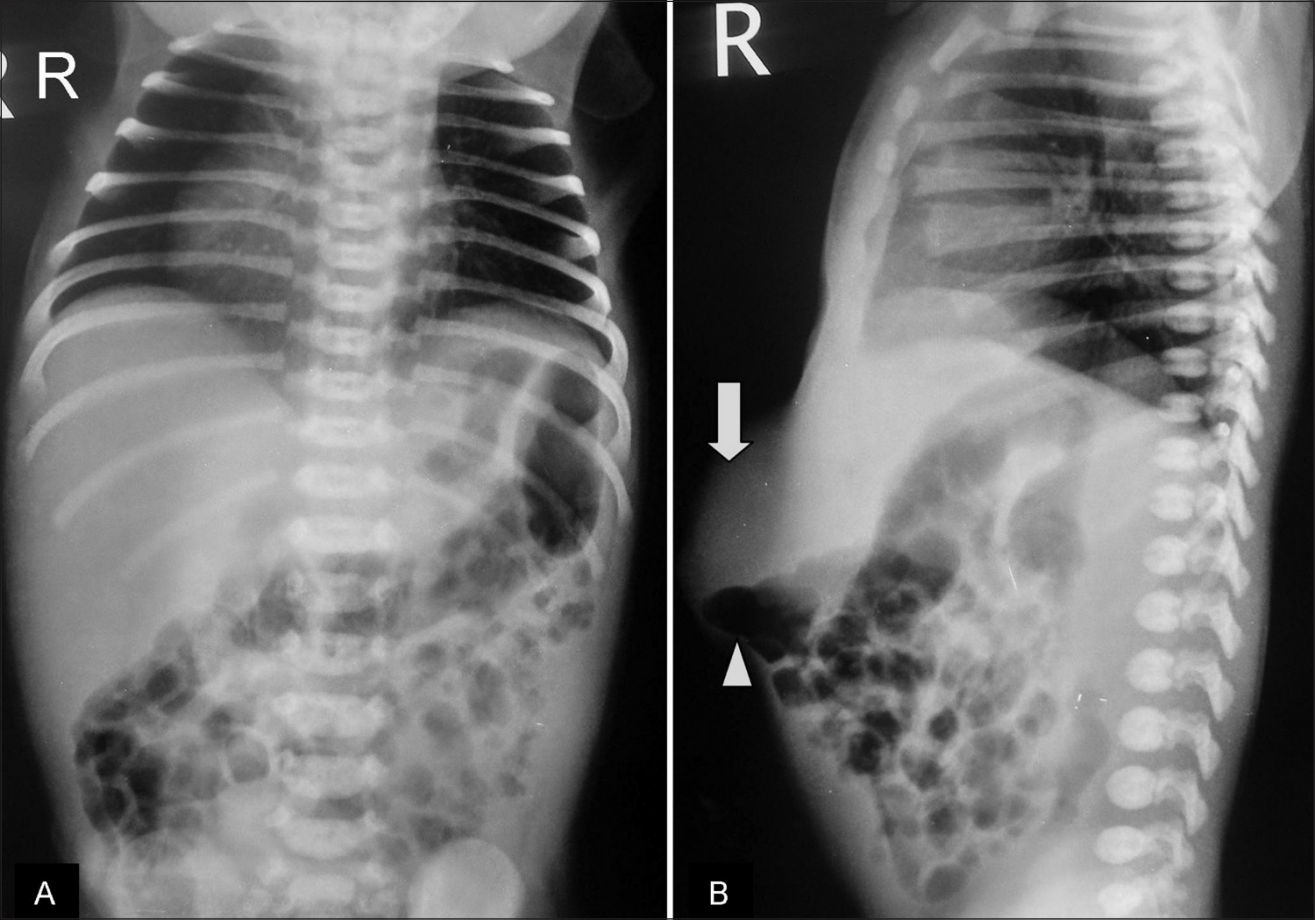
Case 1: A frontal radiograph of the chest (A) shows a dextrorotated heart and reduced pulmonary vascular markings. A lateral radiograph (B) shows a soft tissue swelling (arrow) with air-filled bowel loops (arrowheads) in the umbilical and epigastric region

A chest radiograph at birth showed peripheral pruning of pulmonary vascular markings and a dextrorotated heart [Figure 1].

**Figure 2 (A,B):**
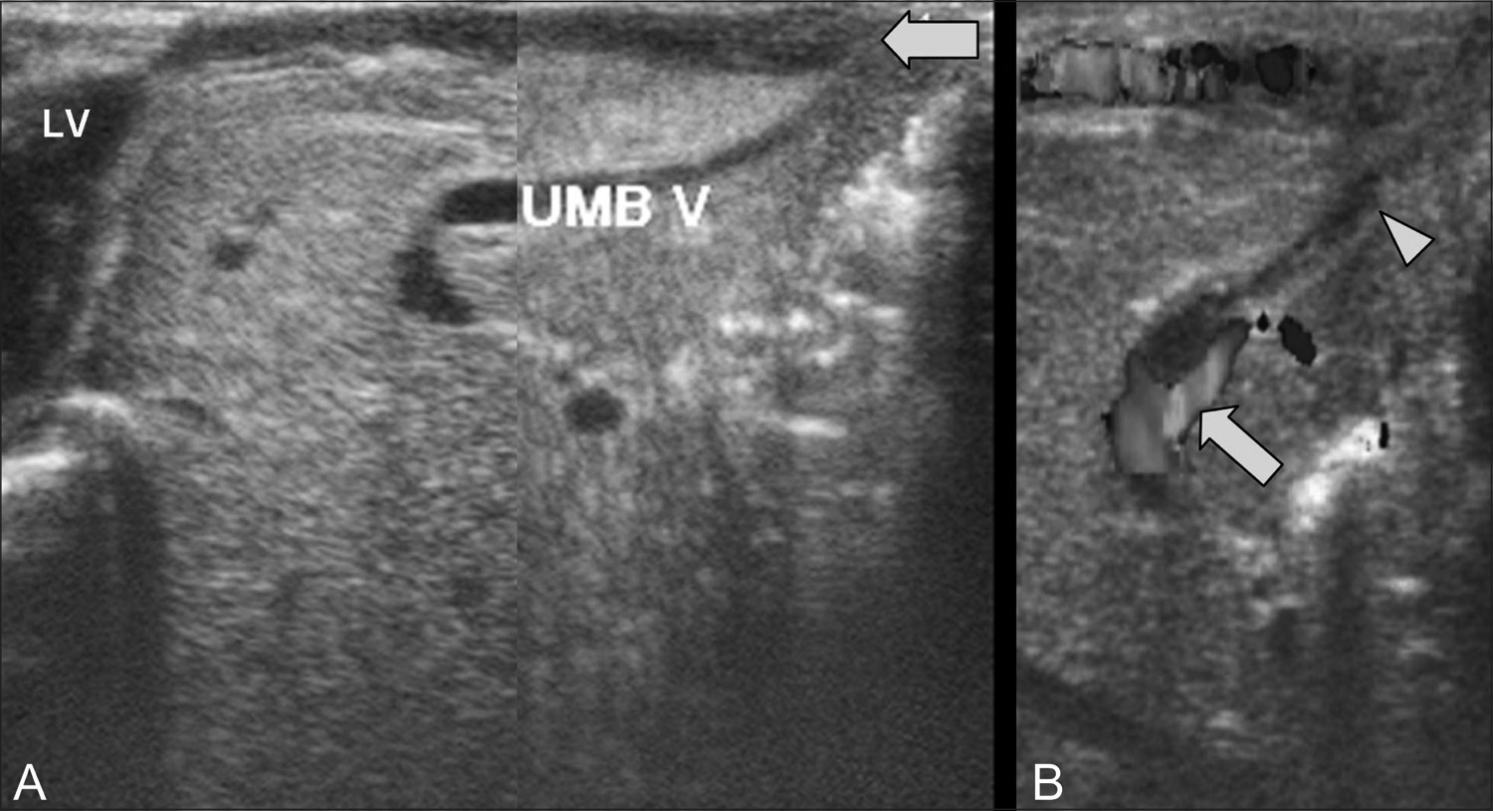
Case 1: A mid-sagittal USG (A) shows a thick tubular channel (arrow) arising from the cardiac apex and extending up to the umbilical region. The flow ceases just above the level of the umbilicus. Color Doppler USG (B) shows communication with the left portal vein (arrow) through the obliterated umbilical vein (arrowhead)

USG of the abdomen showed a thick-walled vascular channel arising from the left ventricular apex; it was seen extending up to the umbilicus, which showed arterial pulsations in concordance with ventricular contractility [Video 1]. Communication with the portal system was present through the obliterated left umbilical vein, which did not show color flow [Figure 2]. A wide-necked abdominal defect was seen at the umbilical region containing bowel loops and the liver. The relationships of the portal vein, the hepatic veins, and the inferior vena cava (IVC) with the right heart were normal.

Under mild sedation, a non-gated CT angiogram was performed using a 64-channel, multidetector CT scanner. The parameters used were as follows: 120 kV, 100 mA, scan delay of 2 seconds and total scan duration of 3 minutes; an automated bolus-tracking technique was used. To minimize radiation to the newborn, only a contrast angiogram study was done.

**Figure 3 (A-D):**
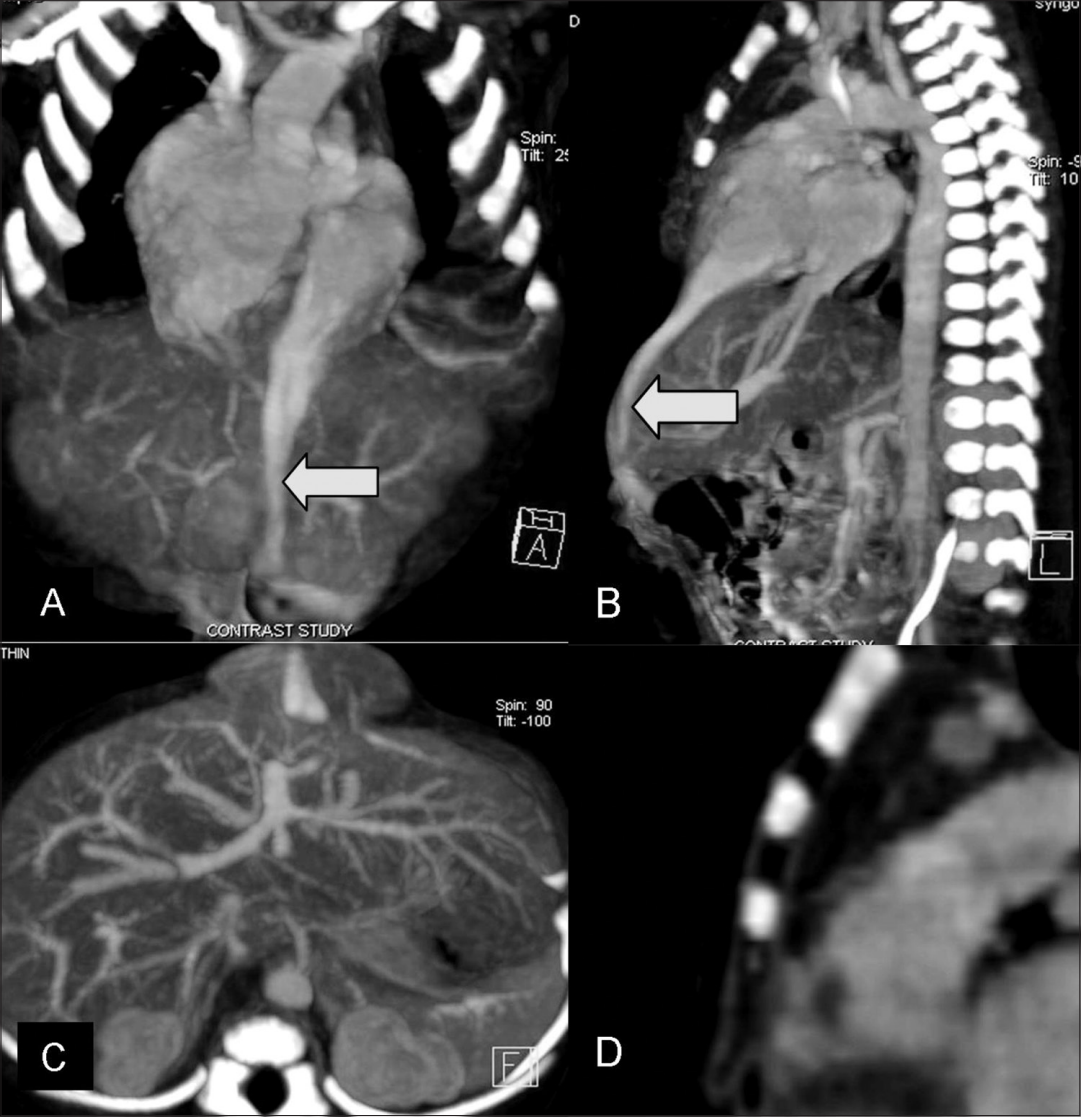
Case 1: Thin coronal (A) and sagittal (B) multiplanar (MPR) CT scan images show a tubular channel (arrow) arising from the cardiac apex and extending up to the umbilical region. The dextraposed aorta is seen arising from the right ventricle. A narrow pulmonary artery can be seen arising from the right ventricle. The diverticulum arises from the cardiac apex. Axial CT scan (C) shows an umbilical hernia containing the left lobe of the liver and the ventricular diverticulum. Sagittal CT scan reconstruction (D) shows the sternum with four ossification centers

**Figure 4 (A,B):**
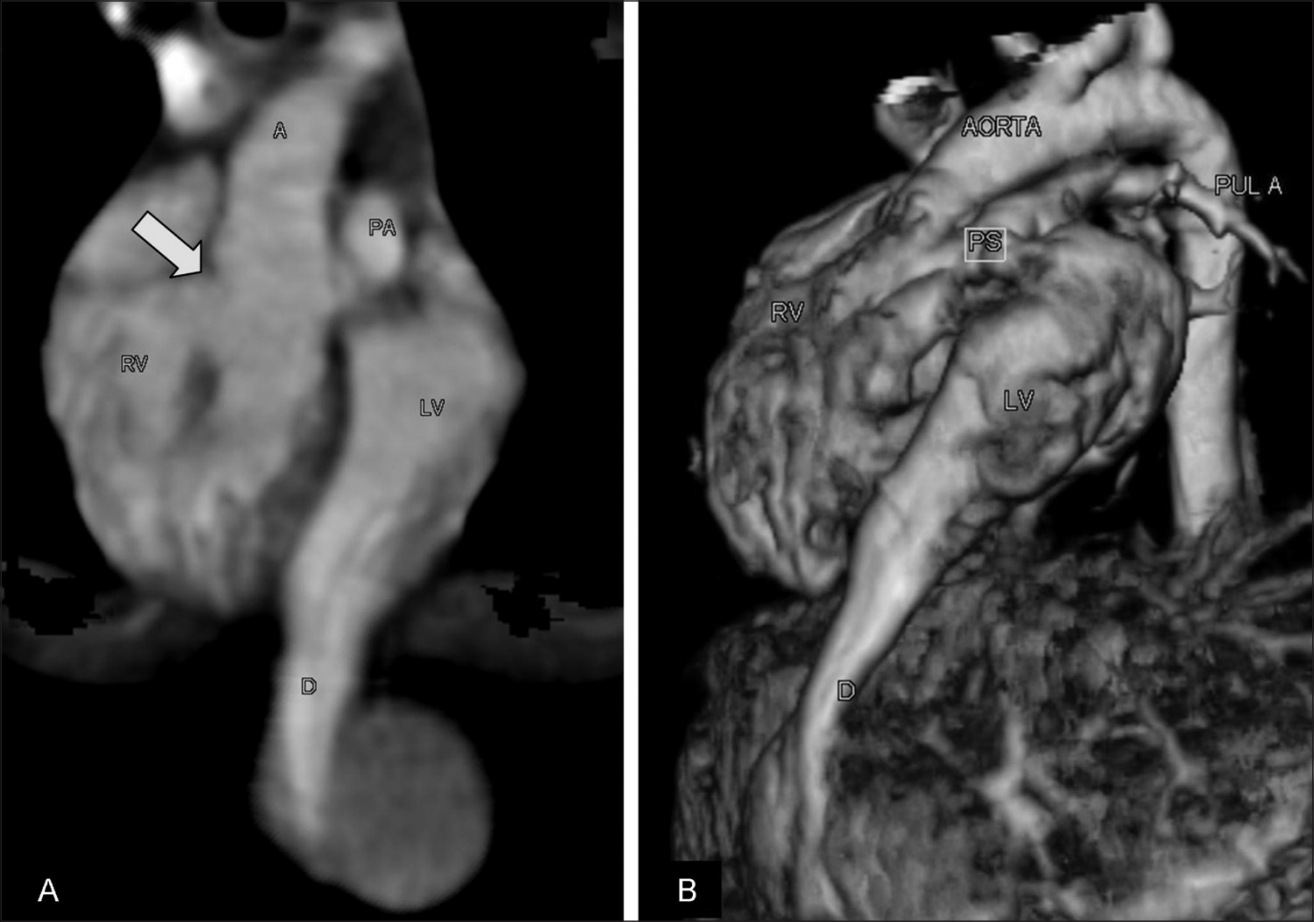
Case 1: A thin overlapping coronal oblique maximum intensity projection (MIP) CT scan image (A) shows the right ventricular outlet (arrow). A volume-rendered 3DCT reconstruction (B) shows the double outlet right ventricle (RV), narrow pulmonary artery (PS), and diverticulum (D) from the cardiac apex

**Figure 5:**
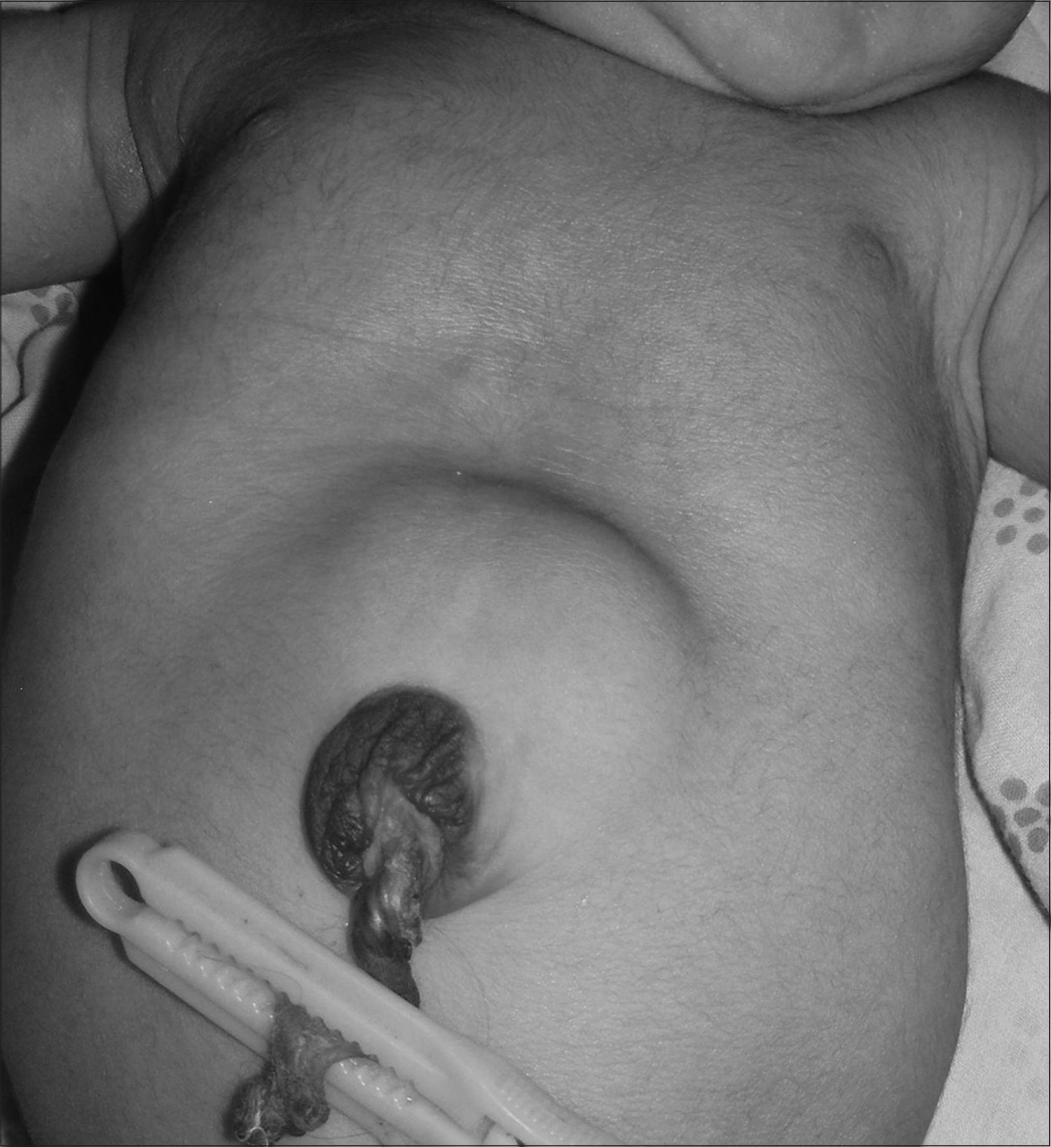
Case 1: Photograph of the baby shows a local swelling at the umbilical region

The angiogram showed a large tubular contrast-filled channel arising from the left ventricular apex [Figure 3], narrowing as it approached the umbilicus. Both the pulmonary artery and the aorta could be seen arising from the right ventricle. The pulmonary artery seemed narrow at its origin [Figure 4]. Herniating bowel and the left lobe of the liver could be seen at the site of the swelling in the umbilical region [Figure 3]. The sternum was normal and showed four sternal ossification centers [Video 2]. Figure 5, a photograph of the baby, shows the swelling in the umbilical and supra-umbilical region.

Case 2

A 15-day-old girl was referred for investigation of an umbilical swelling and epigastric pulsations that had been present since birth.

The baby was the first child of normal parents and there was no significant antenatal history. Examination showed a well-looking infant with epigastric pulsations.

USG showed a thick tubular channel with pulsatile flow in the anterior abdominal wall, posterior to the abdominal musculature; the channel extended in the midline from the left ventricle apex in the xiphisternal region up to the umbilicus.

**Figure 6 (A,B):**
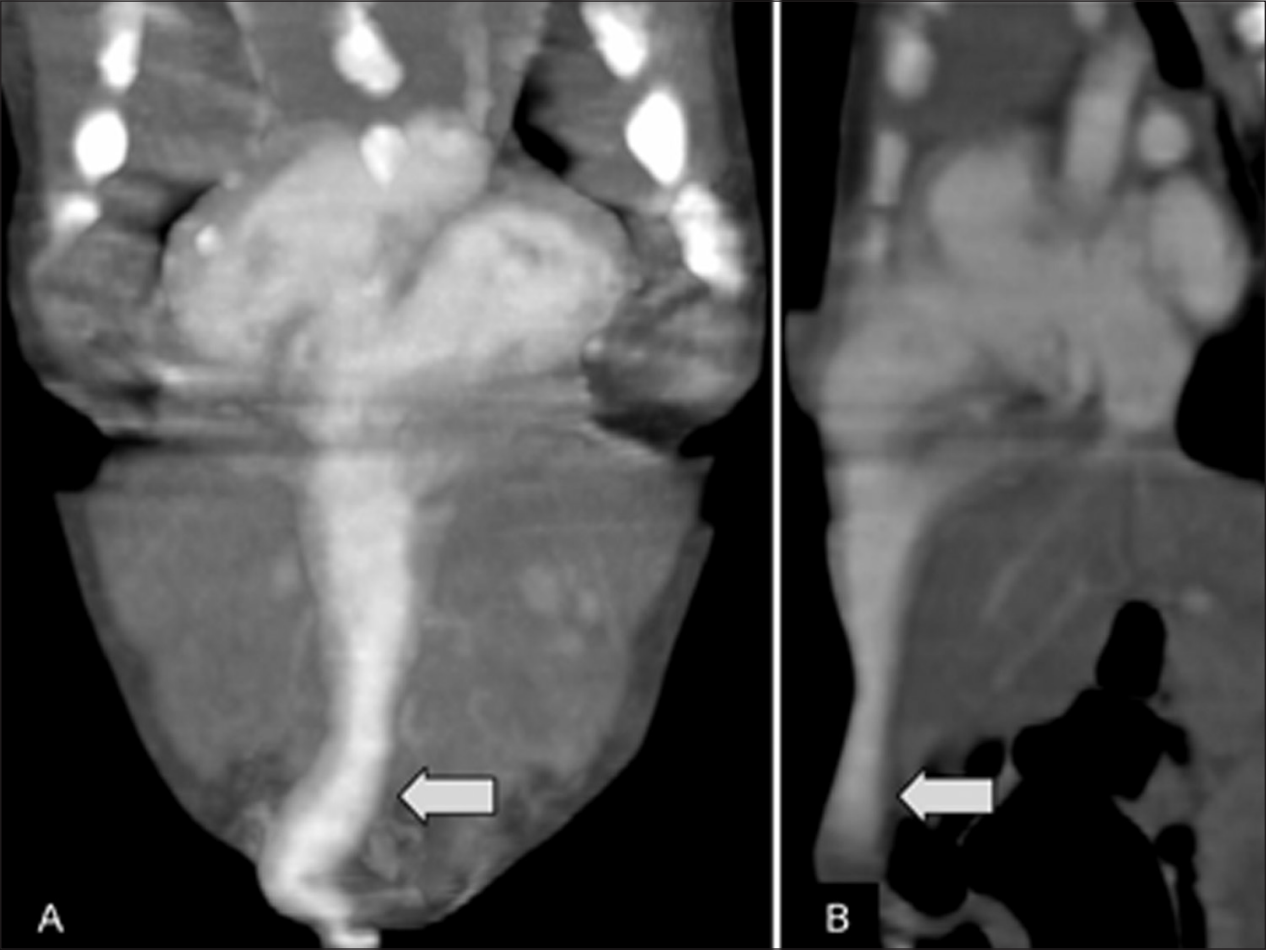
Case 2: Coronal (A) and mid-sagittal (B) thin MIP CT scans show a diverticulum (arrow) arising from the left ventricular apex

**Figure 7 (A,B):**
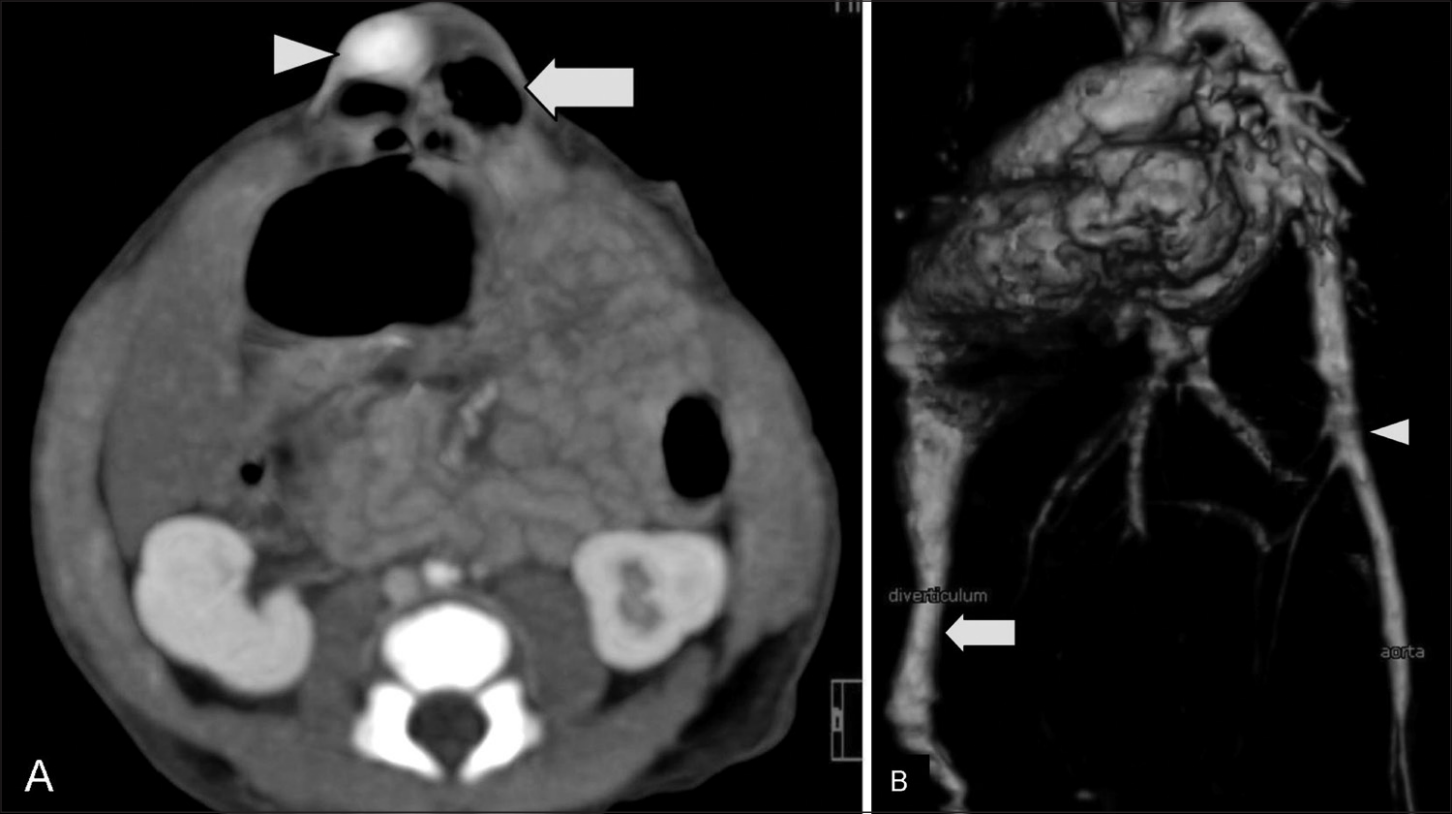
Case 2: Axial CT scan (A) shows umbilical bowel herniation (arrow) and the diverticulum (arrowhead). Volume-rendered 3DCT (B) shows the diverticulum (arrow) on the left and the aorta (arrowhead) on the right

A contrast-enhanced CT scan of the thorax and upper abdomen showed an elongated, tubular, thick-walled structure extending from the left ventricle downward and medially across the diaphragm up to the umbilicus, where it ended blindly [Figure 6]. A midline hernia with herniation of small bowel loops was seen in the umbilical region [Figure 7].

The child died in the hospital due to bilateral bronchopneumonia that was unresponsive to antibiotics and oxygen therapy.

## Discussion

Congenital left ventricular diverticulum starts in the 4th embryonic week. Possible etiologies are intrinsic abnormalities of embryogenesis or in utero acquired malformations (like viral infections, arrhythmia-related vascular accidents or cardiomyopathies).

The first description of a cardiac diverticulum was by Klein in 1953.[1] The prevalence of congenital ventricular diverticulum has been reported to be 0.42% among adult patients undergoing ventriculography after presenting to cardiac clinics with various symptoms.[2]

There have been reports of cardiac diverticulum with pericardial effusion being detected in the second trimester.[3–5] Intrauterine rupture into the pericardium has been offered as the explanation. This is supported by the disappearance of the lesion in postnatal life following therapeutic intrauterine pericardiocentesis.[4,6] Detection of the diverticulum may be incidental or it may be due to related symptoms like embolus, arrhythmia,[7] or infective endocarditis.

The diverticulum may be apical (70%)[8] or non-apical. Most of the apical diverticula are associated with midline thoracoabdominal defects,[1] umbilical hernia, and complex cardiac abnormalities. Non-apical diverticula arise from the subaortic region,[9] the anterior free wall or, rarely, from both ventricles.[10]

Syndromic association was well described by Cantrell et al. in 1958.[11,12] Congenital defects in Cantrell’s pentalogy include abdominal wall defects, sternal defects (aplasia, cleft sternum), anterior diaphragmatic defects, pericardial defects, and complex cardiac abnormalities.[13] Cardiac abnormalities include VSD (100%), atrial septal defect (53%), pulmonary atresia (35%), ectopia cardis (20%), patent ductus arteriosus, tetrology of Fallot, and diverticulum.[11,14] Associated defects include craniofacial abnormalities; hypoplasia of lung,[15] kidneys,[16] adrenal, and liver;[17] malrotation of gut; and limb defects.[15] Cantrell’s pentalogy is underreported as the association is not constant and there is incomplete expression. Defects in the mesodermal development of the anterior transverse septum of the diaphragm are a probable cause.[14]

Abnormal communication of a diverticulum with the portal system through the obliterated umbilical vein can be associated with complex cardiac abnormalities, especially in trisomy 13, 18, and 21. [18–20] Congenital abnormalities involving the umbilical veins are rarely reported; they include persistent right umbilical vein, abnormal course of the umbilical vein anterior to the liver, connecting with the right atrium, continuation as an internal iliac vein,[18,19,21] absent ductus venosus, and aneurysmal dilatation.

Differentiating a ventricular aneurysm from a diverticulum is of importance in adult patients. A narrow mouth and synchronous contractility characterize a diverticulum. On the other hand, aneurysms show akinesia or paradoxic contractility of the outpouching, which is asynchronous with the rest of heart.[22]

A diagnosis can be confidently made with USG and echocardiography. CT angiography, MRI, and invasive ventriculography give a clearer picture of the problem. Functional hemodynamic evaluation can be done and any associated intracardiac complex abnormalities can be detected with echocardiography.

Management of the diverticulum depends on the clinical situation and associated abnormalities. Most asymptomatic diverticula can be managed with a conservative approach. Treatment options for high-risk cases include surgery, anticoagulants, and management of arrhythmias in symptomatic patients.[3]

## See video on www.ijri.org

Click here to view as Video 1

Click here to view as Video 2

## References

[1] Klein G (1953). Casuistics of congenital cardiac diverticulum. Zentralbl Allg Pathol.

[2] Ohlow MA, Secknus MA, Geller JC, von Korn H, Lauer B (2009). Prevalence and outcome of congenital left ventricular aneurysms and diverticula in an adult population. Cardiology.

[3] Perlitz Y, Mukary M, Lorber A, Ben-Ami M (2009). Prenatal diagnosis of fetal cardiac right ventricular diverticulum disappearing at three months of age. A case report and literature review. Fetal Diagn Ther.

[4] Abi-Nader K, David AL, Yates R, Pandya P (2009). Successful outcome after prenatal treatment of a cardiac diverticulum with massive pericardial effusion. Fetal Diagn Ther.

[5] McAuliffe FM, Hornberger LK, Johnson J, Chitayat D, Ryan G (2005). Cardiac diverticulum with pericardial effusion: Report of two new cases treated by in-utero pericardiocentesis and a review of the literature. Ultrasound Obstet Gynecol.

[6] Prefumo F, Bhide A, Thilaganathan B, Carvalho JS (2005). Fetal congenital cardiac diverticulum with pericardial effusion: Two cases with different presentations in the first trimester of pregnancy. Ultrasound Obstet Gynecol.

[7] Pradhan M, Dalal A, Kapoor A, Kumar S, Manisha R (2008). Fetal left ventricular diverticulum presenting as dysrhythmia: diagnosis and management. Fetal Diagn Ther.

[8] Gowitt GT, Zaki SA (1988). Rupture of a cardiac diverticulum resulting in sudden death. Am J Forensic Med Pathol.

[9] Deng Y, Sun Z, Dong N, Du X (2006). Congenital cardiac diverticulum in the subaortic valve area. J Thorac Cardiovasc Surg.

[10] Maroto C, Maroto E, García EJ, Vallés P, Delcán JL, Arcas R (1991). Congenital cardiac diverticulum originating from both ventricles. Rev Esp Cardiol.

[11] Knight L, Neal WA, Williams HJ, Huseby TL, Edwards JE (1976). Congenital left ventricular diverticulum: Part of a syndrome of cardiac anomalies and midline defects. Minn Med.

[12] El Kettani NE, Dafiri R (2006). Isolated congenital left ventricular diverticulum: Report of a paediatric case. J Radiol.

[13] Cantrell JR, Haller JA, Ravitch MM (1958). A syndrome of congenital defects involving the abdominal wall, sternum, diaphragm, pericardium, and heart. Surg Gynecol Obstet.

[14] Edgett JW, Nelson WP, Hall RJ, Fishback ME, Jahnke EJ (1969). Diverticulum of the heart: Part of the syndrome of congenital cardiac and midline thoracic and abdominal defects. Am J Cardiol.

[15] Halbertsma FJ, van Oort A, van der Staak F (2002). Cardiac diverticulum and omphalocele: Cantrell’s pentalogy or syndrome. Cardiol Young.

[16] Yuan SM, Shinfeld A, Mishaly D (2008). An incomplete pentalogy of Cantrell. Chang Gung Med J.

[17] Toyama WM (1972). Combined congenital defects of the anterior abdominal wall, sternum, diaphragm, pericardium, and heart: A case report and review of the syndrome. Pediatrics.

[18] Hajdú J, Marton T, Kozsurek M, Pete B, Csapó Z, Beke A (2008). Prenatal diagnosis of abnormal course of umbilical vein and absent ductus venosus: Report of three cases. Fetal Diagn Ther.

[19] Jouk PS, Champetier J (1991). Abnormal direct entry of the umbilical vein into the right atrium: Antenatal detection, embryologic aspects. Surg Radiol Anat.

[20] Matias A, Huggon I, Areias JC, Montenegro N, Nicolaides KH (1999). Cardiac defects in chromosomally normal fetuses with abnormal ductus venosus blood flow at 10-14 weeks. Ultrasound Obstet Gynecol.

[21] Nakstad B, Smevik B (2004).

[22] Fernández MS, López A, Vila JJ, Lluna J, Miranda J (1997). Cantrell’s pentalogy: Report of four cases and their management. Pediatr Surg Int.

